# Outcomes of Ureteroscopy and Laser Stone Fragmentation (URSL) for Kidney Stone Disease (KSD): Comparative Cohort Study Using MOSES Technology 60 W Laser System versus Regular Holmium 20 W Laser

**DOI:** 10.3390/jcm10132742

**Published:** 2021-06-22

**Authors:** Amelia Pietropaolo, Thomas Hughes, Mriganka Mani, Bhaskar Somani

**Affiliations:** Department of Urology, University Hospital Southampton, Southampton SO16 6YD, UK; ameliapietr@gmail.com (A.P.); t.hughes@doctors.org.uk (T.H.); mrigankamani@gmail.com (M.M.)

**Keywords:** kidney calculi, ureteroscopy, laser, RIRS, Moses, holmium

## Abstract

Background: For ureteroscopy and laser stone fragmentation (URSL), the use of laser technology has shifted from low power to higher power lasers and the addition of Moses technology, that allows for ‘fragmentation, dusting and pop-dusting’ of stones. We wanted to compare the outcomes of URSL for Moses technology 60 W laser system versus matched regular Holmium 20 W laser cases. Methods: Prospective data were collected for patients who underwent URSL using a Moses 60 W laser (Group A) and matched to historical control data using a regular Holmium 20 W laser (Group B), performed by a single surgeon. Data were collected for patient demographics, stone location, size, pre- and post-operative stent, operative time, length of stay, complications and stone free rate (SFR). Results: A total of 38 patients in each group underwent the URSL procedure. The stones were matched for their location (17 renal and 11 ureteric stones). The mean single and cumulative stone sizes (mm) were 10.9 ± 4.4 and 15.5 ± 9.9, and 11.8 ± 4.0 and 16.5 ± 11.3 for groups A and B, respectively. The mean operative time (min) was 51.6 ± 17.1 and 82.1 ± 27.0 (*p* ≤ 0.0001) for groups A and B. The initial SFR was 97.3% and 81.6% for groups A and B, respectively (*p* = 0.05), with 1 and 7 patients in each group needing a second procedure (*p* = 0.05), for a final SFR of 100% and 97.3%. While there were 2 and 5 Clavien I/II complications for groups A and B, none of the patients in group A had any infection related complication. Conclusions: Use of Moses technology with higher power was significantly faster for stone lithotripsy and reduced operative time and the number of patients who needed a second procedure to achieve a stone free status. It seems that the use of Moses technology with a mid-power laser is likely to set a new benchmark for treating complex stones, without the need for secondary procedures in most patients.

## 1. Introduction

The prevalence of kidney stone disease (KSD) has increased worldwide with a lifetime risk in Europe of up to 14% [1]. Ureteroscopy and laser stone fragmentation (URSL) has also seen a big rise over the last two decades [2]. This is attributed partly to the wide availability of holmium:YAG (Ho:YAG) laser systems since its introduction for laser lithotripsy in 1992 [3]. URSL is the first line treatment for large ureteric stones and renal stones up to 2 cm [4,5]. Compared to low power laser lithotripsy, high power lasers seem to require shorter operative time for similar outcomes [6]. The modern high powered Ho:YAG lasers can be equipped with Moses technology, which divides the laser pulse into two peaks. The first pulse separates the fluid in front of the stone (Moses effect), and the second pulse is delivered directly to the stone unimpeded by the intervening fluid, leading to better fragmentation, lower retropulsion and less time taken for the procedure [7,8,9].

Previous in vitro work with Moses technology has shown it to deliver increased stone ablation in soft stones when in contact and 1 mm from the stone [9]. Another in vitro study showed Moses mode to reduce stone movement by 50 times at 0.8 J and 10 Hz, which was seen in both fragmentation and dusting settings [10]. They carried out their work in porcine kidneys to show less ureteral damage on histological analysis after direct lasering of soft tissue, thereby offering safer lithotripsy in shorter time.

The use of laser technique has also evolved and includes dusting, popcorning and pop-dusting [11,12]. The use of laser technology has also shifted from low power to higher power lasers and the addition of Moses technology, which allows for ‘fragmentation, dusting and pop-dusting’ of stones [12]. With high power laser, higher frequency and long pulse allow for the latter, a technique which is now used for large stone treatment in a single setting [12]. A recent systematic review showed that, while high power lasers were faster, this advantage was lost for larger stones [6]. We wanted to compare the outcomes of URSL for Moses technology 60 W laser system versus matched regular Holmium 20 W laser cases. Our hypothesis was that the Moses 60 W laser would achieve better outcomes than the smaller 20 W laser system.

## 2. Methods

Our ureteroscopy outcome audit was registered with ‘Clinical Effectiveness and Audit’ department of our hospital and patient consent was taken for this purpose. Patient outcomes were collected prospectively and recorded in our database, which was then analyzed retrospectively for patient demographics, stone parameters, pre-operative assessment, operative details, laser system used, stone-free rate (SFR), length of stay (LoS), and complication rates.

Patients underwent URSL for ureteral and renal stones using a Moses 60 W laser (Group A) matched to historical control data using a regular Holmium 20 W laser (Group B), performed by a single surgeon (BS) and analysed by a third party (TH) not involved in the original procedure. Patients in Groups A and B had their procedure between March 2012 and May 2014, and August 2019 and October 2020, respectively. LoS was defined from completion of URS to their discharge, with ‘day case’ defined as patients who went home the same day as their surgery [13]. Data were recorded in a Microsoft Excel 2016 (Microsoft, Redmond, WA, USA) and analysed using SPSS version 26 (IBM, Armonk, NY, USA). The independent *t*-test, Mann–Whitney-U test, Chi-squared, and Fisher’s exact test were used with a *p*-value of <0.05 considered statistically significant.

### 2.1. Pre-Operative Assessment

The diagnosis of stone was made on non-contrast CT (CTKUB) for adults and ultrasound (USS) for paediatric patients (<16 years). Positive pre-operative urine culture was treated appropriately based on the sensitivity analysis. All patients also had pre-assessment in dedicated anaesthetic led clinics.

### 2.2. Surgical Technique

A pre-surgical brief was done on the day as per the World Health Organisation (WHO) checklist with the theatre and recovery team where a clear plan was made regarding antibiotic prophylaxis, venous thromboembolism (VTE) prophylaxis and any anticipated surgical or anaesthetic issues.

A protocol-based procedure was done for all patients under general anaesthetic. After initial cystoscopy and safety wire placement, a rigid URS was done using 4.5 or 6F Wolf or Storz semi-rigid ureteroscope over a working wire. For renal stones, based on surgeon discretion, a ureteral access sheath (UAS) was used (9.5F/11.5F or 12F/14F Cook Flexor sheath). A flexible ureteroscopy (Storz FlexX2) and laser (Lumenis, Ltd. Yokneam, Hakidma, Israel) stone treatment was then done using a Moses P60W laser (Group A) or Holmium 20 W laser (Group B). The laser setting used was 0.4–0.8 J, 20–35 Hz with Moses setting for group A and 0.4–0.8 J, 12–18 Hz for group B. Fragments were retrieved using Cook Ngage stone extractor (Cook Medical, Bloomington, IN, USA), with a 6F ureteral stent placed post-operatively when indicated.

### 2.3. Post-Procedural Outcomes

SFR was defined as complete clearance of stones endoscopically and ≤2 mm fragments on post-operative imaging done 2–4 months later. While radiopaque stones were followed up with a plain radiograph, radiolucent stone follow-up was done using an ultrasound scan. If ambiguity remained and patients had symptoms, a CT scan was then done. All intra and post-operative complications were recorded, the latter classified as per the Clavien–Dindo classification system.

## 3. Results

A total of 76 patients (38 patients in each group) underwent a URSL procedure (Table 1). The stones were matched for their location with 17 renal and 11 ureteric stones in each group. The mean age for groups A and B were 53.8 ± 5.8 and 58.1 ± 14.5 years, respectively, with a male:female ratio of 21:17 and 25:13 in the two groups.

The mean single and cumulative stone sizes (mm) were 10.9 ± 4.4 and 15.5 ± 9.9, and 11.8 ± 4.0 and 16.5 ± 11.3 for groups A and B, respectively, with 10 and 9 patients having multiple stones. The pre and post-operative stent rates were 26.3% and 34.2%, and 86.8% and 97.3% for groups A and B, respectively. The mean operative time (min) was 51.6 ± 17.1 and 82.1 ± 27.0 (*p* ≤ 0.0001) for groups A and B. The SFR was 97.3% and 81.6% for groups A and B, respectively (*p* = 0.05), with 1 and 7 patients in each group needing a second procedure (*p* = 0.05), for a final SFR of 100% and 97.3% in both the groups. While there were 2 (5.2%) and 5 (13.1%) complications for groups A and B, none of the patients in group A had any infection related complication. The complications in group A related to stent pain (*n* = 2), and group B related to urosepsis (*n* = 2), urinary tract infection (*n* = 2) and pyelonephritis (*n* = 1).

## 4. Discussion

### 4.1. Meaning of the Study

This study is one of the first to use 60 W Moses Ho:YAG laser in the clinical setting. When compared to the 20 W laser, it was 57% faster (51.6 min versus 82.1 min, *p* < 0.0001) for comparable mean cumulative stone sizes of over 15 mm for both groups. A second procedure was needed for 1 and 7 patients, respectively, for groups A and B (*p* = 0.05) for achieving a SFR of 100% and 97.3%, suggesting a better first-time stone clearance with the 60 W Moses laser. Although not statistically significant, none of the patients in group A had infection related complication compared to 5 in group B. The latter group also had a slightly higher rate of pre- and post-operative stent usage.

### 4.2. Role of Moses Technology and High-Power Laser

Recently, a number of studies have shown the advantage of using both Moses technology and high-power laser for stone fragmentation (6,7,12). With the use of dusting and pop-dusting techniques, large stones (≥15 mm) were treated with a mean operative time of 51 min and an initial SFR of 93% [12]. Using a 120 W generator with 200 micron fiber, a randomised clinical trial (RCT) compared Moses versus regular mode laser lithotripsy for 72 patients. While the total energy and lasing times were similar, Moses mode was associated with significantly less retropulsion (*p* = 0.01), fragmentation/pulverization time (*p* = 0.03) and procedural time (*p* = 0.03) [7]. A recent study comparing 120 W laser with and without Moses mode for benign prostate hyperplasia (BPH) showed better haemostasis and same day discharge with the former [14].

### 4.3. Emerging Advancements in Laser Techniques and Technology

From the early stages of low powered laser lithotripsy, there is now increasing reliance on high power lasers with pulse modulation and newer techniques of fragmentation [15]. The Moses technology has been shown to increase fragmentation and reduce retropulsion. There is now emergence of a thulium fiber laser (TFL) which allows improvements in ablation efficiency and retropulsion with the added advantage of portability, and while more clinical studies need to be done, it has increased the playing field of the laser market giving more choice to the endourologists [16]. Recently, a study from Russia showed the efficacy of TFL for ureteral stones with the authors recommending a setting of 0.5 J, 30 Hz for fragmentation and 0.15 J and 100 Hz for dusting [17]. Another study for TFL on 50 patients showed the safety and efficacy for both ureteral and renal stones [18].

### 4.4. Strengths, Limitations and Areas of Future Research

While this study comes from a single centre and surgeon, it is limited by the retrospective nature of the study design. Apart from the laser, all equipment, techniques and armamentarium used were exactly the same in both time periods. All patients followed the same pathway with their pre-operative assessment and post-operative care. A higher incidence of post-operative infectious complications could be partly explained by higher procedural duration in group B, which is a known pre-disposing factor for this [19]. Similarly, although not significant, group B also had patients with slightly larger stones and higher proportion of patients with pre- and post-operative stents and struvite stones, which are all risk factors for infectious complications [20,21]. Nevertheless, there was a higher SFR and lower secondary procedure rates in group B, suggesting the procedural advantage offered by the 60 W Moses technology when compared to the 20 W technology. In a previous study, procedural time saving did not result in an overall cost saving, which was offset by the cost of the Moses technology [22]. However, this study did not factor the cost associated with the need for secondary procedures. The significantly shorter operative time may increase capacity on operating lists, thereby reducing the time patients are required to wait for their operation, which is beneficial to patients given the substantial impact KSD can have on quality of life [23].

Future studies should ideally be designed as an RCT and consider other aspects such as cost and quality of life, with an emphasis on standardising the outcome measure such as SFR and imaging used to achieve it. Ideally, the SFR should be assessed by a CT scan rather than XR or ultrasound scan. While the role of high-power laser in the field of BPH is more defined, it remains uncertain on the level of advantage it gives to stone surgery. Perhaps a more defined cost-analysis on the cost of machine, laser fiber, scope purchase and repair costs, the cost of procedural time and need for secondary procedure would determine the true value offered by the high-power laser and Moses technology [24,25]. Until then, the 60 W Moses laser might offer a trade-off between cost incurred and outcomes achieved for stone procedures.

## 5. Conclusions

The use of Moses technology with higher power was significantly faster for stone lithotripsy and reduced operative time and the number of patients who needed a second procedure to achieve a stone free status. It seems that the use of Moses technology with a mid-power laser is likely to set a new benchmark for treating large stones, bilateral or multiple stones in a single setting, without the need for secondary procedures in most patients. The exact role of different laser technologies and techniques must be defined for ease of understanding and use in clinical practice.

## Figures and Tables

**Table 1:** Patient and procedural details (PUJ—pelviureteric junction, LP—lower pole, MP—mid pole, LP—lower pole, UA—uric acid, COM—Calcium oxalate monohydrate, COD—calcium oxalate dihydrate, CPC—calcium phosphate carbonate, CHP—calcium hydrogen phosphate dihydrate, MAH—magnesium ammonium phosphate).

	MOSES 60 W (Group A)	Holmium 20 W (Group B)	
Number	38	38	
Age mean ± SD (range), years	53.8 ± 5.8, (9–81)	58.1 ± 14.5, (22–84)	*p* = 0.26
Gender: Male:Female	21 (55.3%): 17 (44.7%)	25 (65.7%): 13 (34.3%)	*p* = 0.35
Side: Left: Right: Bilateral	21:16:1	22:15:1	*p* = 0.97
Location			*p* = 0.52
Ureter	11	11	
PUJ:LP:MP:UP	4:8:3:2	9:4:4:1	
Multiple	10	9	
Single stone size (mm) Mean ±SD (range)	10.9 ± 4.4 (4–24)	11.8 ± 4.0 (4–20)	*p* = 0.34
Cumulative stone length (mm) Mean ±SD (range)	15.5 ± 9.9 (4–57)	16.5 ± 11.3 (5–58)	*p* = 0.63
Number of stones mean ±SD (range)	2.0 ± 2.0 (1–11)	1.8 ± 1.3 (1–7)	*p* = 0.51
Pre-op stent	10 (26.3%)	13 (34.2%)	*p* = 0.45
Post-op stent	33 (86.8%)	37 (97.3%)	*p* = 0.20
Ureteral access sheath	22 (57.8%)	21 (55.2%)	*p* = 0.82
Operation time (min) mean± SD (range)	51.6 ± 17.1 (16–90)	82.1 ± 27.0 (40–160)	*p* ≤ 0.0001
Initial Stone Free rate (SFR)	37 (97.3%)	31 (81.6%)	*p* = 0.05
Final SFR	38 (100%)	37 (97.3%)	
Patients requiring 2nd procedure	1 (2.6%)	7 (18.4%)	*p* = 0.05
Length of stay (LOS) (days) median (range)	0 (0–2)	0 (0–6)	*p* = 0.26
Stone analysis			
UA + COM	3	1	
COM	15	7	
COD + COM + CPC	1	1	
COD + CHP + COM	2	1	
COD + CPC	3	1	
COM + COD	2	3	
COM + CPC	8	9	
CPC + MAH	1	6	
Cystine	2	2	
UA	0	1	
Complications (%)	2 (5.2%)	5 (13.1%)	*p* = 0.43
Pain	2	0	Clavien I
Urosepsis	0	2	Clavien II
UTI	0	2	Clavien II
Pyelonephritis	0	1	Clavien II

## Data Availability

Data is available and kept in the hospital electronic system.
